# First look: a cluster-randomized trial of ultrasound to improve pregnancy outcomes in low income country settings

**DOI:** 10.1186/1471-2393-14-73

**Published:** 2014-02-17

**Authors:** Elizabeth M McClure, Robert O Nathan, Sarah Saleem, Fabian Esamai, Ana Garces, Elwyn Chomba, Antoinette Tshefu, David Swanson, Hillary Mabeya, Lester Figuero, Waseem Mirza, David Muyodi, Holly Franklin, Adrien Lokangaka, Dieudonne Bidashimwa, Omrana Pasha, Musaku Mwenechanya, Carl L Bose, Waldemar A Carlo, K Michael Hambidge, Edward A Liechty, Nancy Krebs, Dennis D Wallace, Jonathan Swanson, Marion Koso-Thomas, Rexford Widmer, Robert L Goldenberg

**Affiliations:** 1 Department of Statistics and Epidemiology, RTI International, Durham, NC, USA; 2 Department of Radiology, University of Washington, Seattle, WA, USA; 3 Department of Community Health Sciences, Aga Khan University, Karachi, Pakistan; 4 Department of Pediatrics, Moi University School of Medicine, Eldoret, Kenya; 5 Francisco Marroquin University, Guatemala City, Guatemala; 6 Department of Pediatrics, University Teaching Hospital, Lusaka, Zambia; 7 University of Kinshasa School of Public Health, Kinshasa, Democratic Republic of the Congo; 8 Department of Obstetrics, Moi University School of Medicine, Eldoret, Kenya; 9 FANCAP, Guatemala City, Guatemala; 10 Department of Radiology, Aga Khan University, Karachi, Pakistan; 11 RTI International, Durham, NC, USA; 12 Kinshasa School of Public Health, Kinshasa, Democratic Republic of the Congo; 13 Department of Pediatrics, University of North Carolina at Chapel Hill, Chapel Hill, NC, USA; 14 Department of Pediatrics, University of Alabama at Birmingham, Birmingham, AL, USA; 15 Department of Pediatrics, University of Colorado Health Care System, Denver, CO, USA; 16 Department of Pediatrics, Indiana University School of Medicine, Indianapolis, IN, USA; 17 Eunice Kennedy Shriver NICHD, Bethesda, MD, USA; 18 GE Healthcare, Wisconsin, USA; 19 Department of Obstetrics and Gynecology, Columbia University, New York, NY, USA

**Keywords:** Maternal mortality, Maternal near miss, Perinatal mortality, Obstetric ultrasound, Low-income countries

## Abstract

**Background:**

In high-resource settings, obstetric ultrasound is a standard component of prenatal care used to identify pregnancy complications and to establish an accurate gestational age in order to improve obstetric care. Whether or not ultrasound use will improve care and ultimately pregnancy outcomes in low-resource settings is unknown.

**Methods/Design:**

This multi-country cluster randomized trial will assess the impact of antenatal ultrasound screening performed by health care staff on a composite outcome consisting of maternal mortality and maternal near-miss, stillbirth and neonatal mortality in low-resource community settings. The trial will utilize an existing research infrastructure, the Global Network for Women’s and Children’s Health Research with sites in Pakistan, Kenya, Zambia, Democratic Republic of Congo and Guatemala. A maternal and newborn health registry in defined geographic areas which documents all pregnancies and their outcomes to 6 weeks post-delivery will provide population-based rates of maternal mortality and morbidity, stillbirth, neonatal mortality and morbidity, and health care utilization for study clusters. A total of 58 study clusters each with a health center and about 500 births per year will be randomized (29 intervention and 29 control). The intervention includes training of health workers (e.g., nurses, midwives, clinical officers) to perform ultrasound examinations during antenatal care, generally at 18–22 and at 32–36 weeks for each subject. Women who are identified as having a complication of pregnancy will be referred to a hospital for appropriate care. Finally, the intervention includes community sensitization activities to inform women and their families of the availability of ultrasound at the antenatal care clinic and training in emergency obstetric and neonatal care at referral facilities.

**Discussion:**

In summary, our trial will evaluate whether introduction of ultrasound during antenatal care improves pregnancy outcomes in rural, low-resource settings. The intervention includes training for ultrasound-naïve providers in basic obstetric ultrasonography and then enabling these trainees to use ultrasound to screen for pregnancy complications in primary antenatal care clinics and to refer appropriately.

**Trial registration:**

Clinicaltrials.gov (NCT # 01990625)

## Background

In low-income countries (LIC), maternal mortality rates range from 150 to more than 1000 per 100,000 live births while rates of stillbirth and neonatal mortality range from 20 to 40 per 1000 births [1-4]. Each of these outcomes occurs at least ten times more frequently in LIC than in high income countries (HIC) [5,6]. Maternal ‘near miss’, defined by the World Health Organization (WHO) as women who nearly die but survive a complication during pregnancy, is another outcome measure [7,8]. Near misses, closely related to maternal mortality, may occur as frequently as 33 per 1000 births in LIC [9-11]. Because maternal mortality is relatively rare and therefore difficult to study, many have advocated using “near miss” as a surrogate measure of maternal mortality, although morbidity is an important outcome in its own right [12]. Furthermore, approximately 15% of all pregnancies have medical or obstetric complications that greatly increase the risk of mortality or severe morbidity for the mother and newborn [13-15].

The reasons for the discrepancies in rates between HIC and LIC are many, but factors include lack of antenatal care and limited access to facilities that can provide lifesaving treatment for the mother, fetus and newborn [16]. Universal access to high quality facility care substantially reduces mortality and morbidity from these conditions [17,18]. In HIC, prenatal care is nearly universal, and, as part of that care, obstetric ultrasound is used routinely to determine gestational age and screen for complications including multiple gestations, fetal anomalies, mal-presentation, and placenta previa [19]. In LIC, factors including high cost of the ultrasound equipment, lack of trained sonographers or physicians, as well as the skill required for performing the examinations have been among the barriers to use [20,21].

The inequality in pregnancy outcomes, the global push to accelerate fulfillment of the United Nations Millennium Development Goals (MDGs) 4 and 5 to reduce maternal and child mortality, and reductions in the size and cost of electronics have increased interest in the use of ultrasound to improve pregnancy outcomes in low- and middle-income countries [22]. Increased interest, however, does not imply effectiveness nor a consensus on ultrasound’s ability to improve maternal and newborn health in LIC. Public health academicians often cite a Cochrane review of studies conducted in high-resource settings that concluded that routine late ultrasound (after 24 weeks), excluding patients determined clinically to be high-risk, had no measurable benefit for mother or baby [23]. Regardless, ultrasound examinations are now a routine component of obstetric care in many high income countries and usage is increasing in middle and low income countries [19]. Given the lack of consensus and supportive evidence on the value of ultrasound in improving outcomes by antenatal detection of complicated pregnancies, an authoritative investigation on ultrasound’s utility in LIC settings will be important and timely.

### Previous research

Studies of antenatal ultrasound in LIC settings conducted to date have not been powered to detect differences in maternal and newborn mortality [24,25]. However, several studies found psychological benefit for mothers and increased facility utilization associated with obstetric ultrasounds performed during prenatal care [26-28]. Additionally, clinical competence of paraprofessionals (e.g., midwives, paramedics) to perform obstetric ultrasound [29-34] with a relatively short training period has been shown [35,36]. However, the optimal training regimen to reach competency has not been determined.

A summary of potential pregnancy-related benefits and risks to the use of ultrasound in LIC settings is included in Table 1. In addition to the potential benefits, there are also a number of potential challenges associated with introducing ultrasound in low-resource settings (Table 2). Together, the issues reviewed (Tables 1 and 2) suggest the need for a multi-country trial to evaluate the potential for ultrasound to improve maternal and perinatal outcomes in low-resource settings.

**Table 1:** Potential benefits of obstetric ultrasound in antenatal care

***Diagnoses of the following conditions***	
•	Gestational age to diagnose prematurity and post-dates
•	Placenta previa
•	Fetal malposition
•	Multiple gestation
•	Ectopic pregnancy
•	Retained placental products following delivery
•	Fetal anomalies
•	Fetal growth restriction
•	Poly- and oligio-hydramnios
•	Fetal demise
•	Obstructive fibroids
*Potential benefits related to health care*	
•	Increased enrollment in prenatal care (access to testing for syphilis, iron/vitamins, etc.)
•	Increased basic facility usage for delivery for women with uncomplicated pregnancies
•	Increased hospital referral for delivery for women with complicated pregnancies
•	Decreased inappropriate transfers
•	Recruitment and retention of community physicians and midwives
•	Specific diagnostic information to inform expecting mothers to deliver in a risk-appropriate setting
*Potential outcomes achieved with prenatal ultrasound*	
•	Reduction of maternal mortality and maternal near-miss morbidity
•	Reduction of fetal and newborn mortality
•	Rational management of preeclampsia/eclampsia, fetal growth restriction and other conditions related to gestational age dating
•	The ability to treat women with a short cervix with progesterone or a pessary to decrease preterm birth
•	Appropriate treatment of women with incomplete abortion, ectopic pregnancy, and fetal demise
•	Reduction in emergency care for birthing complications due to more deliveries in risk-appropriate settings

**Table 2:** Challenges and issues related to introduction of ultrasound in low-resource settings

***Potential challenges with the introduction of ultrasound include***	
•	Security of the ultrasound machine
•	Prevention of the use of the ultrasound equipment for sex determination/selection
•	Infrastructure requirements, including electricity and maintenance
•	Training issues for community physicians and para-professionals
•	Diversion of resources from other clinical activities to ultrasound
•	Use of resources required for life saving interventions to expenditures for US equipment
•	Increase in unnecessary interventions
•	Attrition of trained ultrasound personnel
•	Health facilities improving to meet increases in demand generated by ultrasound
•	Sustaining funding for continuous improvement in ultrasound training and care delivery
*Issues related to training various providers to use ultrasound identified include:*	
•	Defining the level of health care personnel who can be effectively trained in ultrasound use.
•	Country regulations for type of health professional allowed to be certified in ultrasound use.
•	Acceptability at the policy level of this trial to train health care professionals other than physicians and sonographers.
•	Defining the type and length of training required to achieve reliable diagnoses by community physicians and non-physicians with various levels of training.
•	Determining how well ultrasound can identify various conditions at different levels of care.
•	Logistics of providing care while essential personnel are in ultrasound training.

## Methods/Design

The cluster-randomized controlled trial (RCT) will be undertaken to address two important hypotheses. The primary hypothesis is that antenatal ultrasound screening at antenatal clinics (ANC) will improve a composite outcome of maternal mortality, maternal near miss, stillbirth, and neonatal mortality. Two important secondary hypotheses (which will be assessed independently without adjustment for multiple comparisons) that the study has been powered to test is that ultrasound will increase 1) the rate of prenatal care utilization and 2) utilization of health facilities for delivery by for women with complicated pregnancies. Additional secondary outcomes include the difference between ultrasound and non-ultrasound clusters in rates of maternal mortality, maternal morbidity, neonatal deaths, intra-uterine growth restriction-related mortality, and intrapartum stillbirths. Because the effectiveness of training is an important consideration for implementation, an additional part of the study is evaluation of the training component.

The trial will be undertaken in five LIC rural settings. The study design is a cluster RCT with intervention and control clusters stratified by country, while also considering factors such as historic perinatal mortality rates and logistic factors such as travel time to Emergency Obstetric and Neonatal Care facilities. Each ultrasound cluster will be defined by a health center and its catchment area and generally have approximately 500 births per year. The clusters will be stratified by country and then randomized to the intervention group or to the control group.

### Study intervention

The main study intervention will be routine ultrasound examinations offered to all intervention cluster pregnant women to encourage utilization of antenatal care, to identify pregnancies with complications and for pregnancies identified with complications, to offer appropriate referral to a higher level of care.

For the intervention clusters, while flexible, the general plan is to develop an ultrasound team consisting of one to two sonographers and an assistant who will serve approximately 3–5 health clinics (or clusters). The field team of sonographers will be overseen by a project sonographer at the referral hospital associated with the intervention cluster. Therefore, if a site has 20 clusters, there will be at least 2 teams of sonographers together serving 10 intervention clusters. An additional 10 control clusters will collect the primary outcome data but will not have active intervention activities.

In intervention clusters, the goal is to provide two routine prenatal screening ultrasound examinations to each pregnant woman living in those clusters. The first ultrasound will generally be performed at 18–22 weeks gestational age to document gestational age, fetal number, fetal position, amniotic fluid abnormalities, cervical length, and major congenital anomalies. A second ultrasound examination will be performed at 32–36 weeks to determine placenta location and growth in addition to gestational age, fetal position, amniotic fluid abnormalities, cervical length, and major congenital anomalies. Based on the results of these ultrasound examinations, mothers will be counseled about the need to seek care or deliver at a referral hospital. If a women presents to the clinic with a complication and she is outside the window for a screening examination, at the discretion of the clinic staff, she may receive an ultrasound examination and be counseled based on its results. Because providing a picture of the fetus may contribute to increased clinic utilization, each of the ultrasound machines will be provided with a printer so that pictures of the fetus can be provided to the mother at each visit.

### Trial organization and sites

Funded by the Bill & Melinda Gates Foundation, the ultrasound trial will be conducted under the auspices of the Global Network for Women’s and Children’s Health Research (Global Network), a multi-country research network supported by the *Eunice Kennedy Shriver* National Institute of Child Health and Human Development. The University of Washington, Department of Radiology (UW) will oversee the obstetric ultrasound training with support from GE Healthcare. A study subcommittee comprised of Global Network investigators and UW faculty will oversee the study, with *ex officio* representation from funders.

### Ethics approval

The study protocol has been reviewed and approved by ethics review committees including the University of Washington (Seattle, WA), Columbia University (NY, NY), Research Triangle Institute (Durham, NC), University of Zambia (Lusaka, Zambia), Kinshasa School of Public Health (Kinshasa, DRC), Moi University (Eldoret, Kenya), Aga Khan University (Karachi, Pakistan), and Francisco Marroquin University (Guatemala City, Guatemala).

The study sites are clusters in rural areas in Chimaltenango, Guatemala, Lusaka District, Zambia, Equateur Provence, Democratic Republic of Congo; Western Provence, Kenya, and Thatta District, Pakistan. In these areas, over half of the births occur at home (Table 3). The trial will use the Global Network’s Maternal Newborn Health (MNH) pregnancy registry to collect outcome data, as described elsewhere [37]. Based on the sample size described below, we plan to implement the study in a total of 58 clusters (29 intervention and 29 control).

**Table 3:** Ultrasound trial sites

	**Chimaltenango Guatemala**	**Lusaka Zambia**	**Western Provence Kenya**	**Thatta Pakistan**	**DRC**
Study Clusters* (N)	18	10	12	10	8
Births 2009-2010** (N)	10,706	14,154	17,541	25,909	NA
**Birth attendant (%)**					
Physician	27.9	2.7	1.6	22.7	0.1
Nurse/midwife	1.5	43.9	34.8	25.1	21.3
TBA	70.4	32.2	51.1	49.7	77.5
Family/unattended	0.2	21.2	12.5	2.5	12.1
**Birth location (%)**					
Hospital	26.0	5.7	9.5	24.3	0.1
Health clinic	3.1	42.0	25.6	23.3	25.4
Home	70.9	52.2	64.9	52.3	74.5
**C-section rate (%)**	11.4	1.0	1.1	6.6	0.1
**Mortality rates/1000**					
Neonatal (28 day) **	27	22	16	45	27
Stillbirth	22	27	20	54	23
Maternal mortality ratio/ 100,000, Mean	95	211	88	239	540

*Study clusters for the Ultrasound Trial.

**DRC birth rates based on 2006–2007 and 7-day neonatal mortality rate [38].

In the ultrasound trial intervention clusters, an ultrasound examination will be offered to pregnant women at ANC visits at 18–22 and at 32–36 weeks using the sonographers trained for this project. In addition, in the intervention clusters there will be additional provider training for obstetric emergencies at the referral hospitals and community notification about the project and referral enhancement (described below). In control clusters, no ultrasound examinations will be provided, there will be no community notification, and there will not be additional training to referral hospitals serving only the control clinics. Data on pregnancy outcomes will be collected in a similar fashion in both the ultrasound control and the ultrasound intervention clusters by MNH Registry personnel.

### Inclusion and exclusion criteria

All pregnant women who present at a study clinic for ANC will be screened for the trial, using the following inclusion and exclusion criteria. Inclusion criteria are women who provide informed consent and are residents of study cluster, enrolled (or eligible) in the Global Network MNH Registry, ≥ 16 weeks gestation at the initial antenatal care visit and who meet the local age criteria to consent to participate. Women who are in labor at time of consent will be excluded.

### Study intervention

#### *Obstetric sonography training*

The two-week obstetric ultrasound training will be provided to non-physician health workers in ultrasound use, diagnosis, and appropriate referral. For each site, the cadre of trainees may vary (e.g., clinical officers, nurses, and nurse midwives) but sufficient numbers will be trained at each site to ensure that sonographers are available at the intervention health centers to provide routine ultrasound during ANC. Training also will be provided to the relevant staff at primary referral facilities to ensure the continuity of health care provision. This ultrasound training will occur under the direction of Dr. Robert Nathan, a radiologist based at UW, utilizing the curriculum, the *Basic Obstetric Ultrasound Training Instructor Guide* and *Basic Obstetric Ultrasound Participant Manual,* developed at UW [39]. Additionally, central trainers will evaluate the skills of each trainee, and certify trainees who successfully complete the course to participate in the trial using standardized pre-post written and scanning tests. A sonographer at a local referral hospital will be trained to provide ongoing training, quality assurance (QA), and evaluation of each trainee. Data will be collected to evaluate trainee competence and ongoing training needs.

#### *Pilot period and ongoing QA*

We will include a 3-month pilot period following the initial sonography training to ensure that the trial procedures have been implemented. The pilot period will evaluate the sonographer’s ability to obtain adequate exams, the integration of the ultrasound team into the antenatal care clinic, the number of ultrasound exams possible, the effectiveness of the referral system, the ability of the referral hospitals to care for those referred, and the ability of the ultrasound team to collect and record accurate data.

Following the pilot period, the ongoing QA systems will include upload of US images on a project website for QA purposes and periodic live reviews of community sonographer imaging in the field. The UW sonography team, together with the designated referral facility obstetrician or radiologist, will similarly review the effectiveness of the sonographers at the referral facilities and recommend actions to establish and maintain a level of imaging proficiency.

Finally, for the QA process, each site will hold a refresher sonography training session (approximately 2–3 days in duration) approximately 6 months following the initial 2-week sonographer training. The refresher training objectives are to evaluate the retention of trainee skills to identify and address areas of weakness, and to ensure understanding of the obstetric skills. This training provides an opportunity to reinforce the study objectives and strengthen relationships with the referral hospital sonographers.

#### *Emergency obstetric and neonatal care training*

The second component of the intervention will be training of referral hospital staff in management of obstetrical conditions. This will be performed at each site by a project obstetrician with skills in in-hospital training to provide care for the major obstetric/neonatal conditions such as preeclampsia/eclampsia, obstetric hemorrhage, and newborn resuscitation. Obstetric/neonatal drills in the management of obstetric/neonatal conditions will play an important role in this training. The training will be concentrated in the initial 3 months, but we will plan for continual evaluation of hospital function and ongoing training to improve deficiencies noted.

#### *Referral and system enhancement*

This component will focus on working with ministry of health officials and hospital administrators in improving health system management, especially since integration of appropriate referrals between the health clinics and the hospitals will be a component of ultrasound training. While this will not be a major trial component, we expect to hold several sessions with appropriate health system leaders and administrators to discuss integration of obstetric/neonatal care between the primary health clinics and the referral hospitals.

#### *Community sensitization*

Finally, we will hold community orientations in each location where ultrasounds will be available to improve awareness of the service, when and where it will be offered, and the conditions ultrasound can diagnose. This activity will also determine and address barriers to receiving ultrasound at the community level. Depending on the community structure, the community notification portion of the intervention may overlap with some of the referral and transfer system analyses described above. For example, meetings in the intervention clusters may include government officials and hospital and health center administrators, obstetric providers (physicians, nurse midwives, auxiliary nurse midwives), and, where applicable, community or traditional birth attendants, and health workers. It will also, as stated above, include a series of ultrasound intervention cluster village meetings so that women and their families and village elders will understand the trial.

#### *Sample size considerations*

The primary outcome is a composite of four components (stillbirth, neonatal mortality, maternal mortality, and maternal near miss). Within the Global Network, the neonatal rates average about 27 per 1,000 births, the stillbirth rates average about 33 per 1,000, and the maternal mortality is approximately 2 per 1,000 births [37,40,41]. While we have not analyzed Global Network maternal near miss data, studies suggest that for every maternal death there are 5 to 10 near misses and we estimate that the maternal near miss rate within the Global Network is 20 to 30 per 1,000 births. This estimate coincides with several developing country estimates of near misses ranging from 2% to 3% of all pregnancies. For sample size calculations we assumed a near miss rate of 25 per 1,000 births. Thus, our composite outcome baseline rate is estimated to be in the range of 80 to 90 events per 1,000 pregnancies with current care in the trial clusters.

Sample size estimates for potential decreases in the composite maternal, fetal, and neonatal outcomes with 90% power and alpha at 0.05 for rates of the composite outcome in the range of 70 to 90 events per 1000 deliveries, target reductions of 20% to 25%, an intra-cluster correlation (ICC) of 0.005 (a rate that is consistent with historic Global Network mortality rates). Based on the power calculations with these assumptions, 58 clusters will provide more than 80% power to detect a 25% improvement in the composite outcome over a range of assumptions that are reasonable based on historic MNH Registry data; the design will provide 79% to 94% power to detect as little as a 20% reduction over the range of underlying risks.

#### *Data analyses*

The primary analysis, which will utilize data at the cluster level, will be based on an alternative to a classic randomization test which involves a two stage approach [42]. First, a linear model is fit with the cluster as the analysis unit and the composite mortality/morbidity rate for each cluster as the outcome. In the second stage, the residuals from the first stage model and the t-test procedures [42] will be used to generate a hypothesis test of the difference in composite mortality/morbidity and to generate 95% confidence intervals for the difference in this rate between the two treatment arms, controlling for site and randomization stratum.

To facilitate the model-based primary analysis, we will evaluate baseline differences in key demographic and clinical measures between the intervention and control clusters to assess the extent, if any, of baseline imbalances between the two groups that might confound study inferences. If baseline differences are identified, we will include appropriate cluster-level or individual-level variables to evaluate the impact of these variables on the observed treatment effect. Because the primary interest is in the increase in the marginal morbidity/mortality rate at the cluster level, the generalized estimating equations method [26] will be used for the primary analysis. The model will control for cluster-level effects by assuming country-specific within-country correlation within clusters, and all inferences will be based on the empirical correlation matrix [26].

## Discussion

The cluster RCT will evaluate the impact of a community-based intervention focused on introduction of obstetric ultrasound into ANC by community health providers.

While the major intent of the trial is to assess the effect of ultrasound on pregnancy outcomes and appropriate referral, an ultrasound machine is unlikely to achieve benefit without health workers who have appropriate training, continued quality assessment, and oversight. Therefore, an important trial component is training of the health workers to use ultrasound and to identify the conditions that require referral. It is also important that pregnant women and their communities be aware of ultrasound availability. Thus, a community notification and sensitization component will be included to increase awareness of ultrasound and pregnancy complications directed not only at pregnant women, but for husbands, family, and community leaders. Staff will also be trained to explain all ultrasound findings and recommended actions to the patient and her family, and to facilitate referrals for confirmatory ultrasound and additional counseling, as needed.

Having a referral institution with staff trained to review ultrasound findings and manage complications is crucial. At a minimum, the successful introduction of ultrasound into primary health centers and clinics will require an evaluation of the health systems’ capabilities, and discussion with local policy makers and providers to ensure that the referral hospitals can appropriately manage the obstetric complications referred to it.

With the decreasing costs and increasing availability of ultrasound in low-resource settings, understanding the potential impact of ultrasound not only on the health of the mother and her fetus but on the health system is needed. This multi-faceted intervention, which represents a unique partnership of academic and clinical investigators supported through public and private funding, will provide results to inform the issues around ultrasound use.

## Abbreviations

ANC: Antenatal care; CS: Cesarean section; EMONC: Emergency obstetric and neonatal care; HIC: High-income countries; ICC: Inter-cluster correlation; LIC: Low-income countries; RCT: Randomized controlled trial; US: Ultrasound; WHO: World Health Organization.

## Competing interests

The authors declare that they have no competing interests.

## Authors’ contributions

EMM and RLG – Developed the initial protocol concept and wrote the first draft; CLB, DB, WAC, EC, FE, HF, LF, AG, KMH, NK, AL, EAL, DM, HM, WM, MM, SS, MKT and AT participated in the protocol development and reviewed the final draft; RON, JS, DS – Conceptualized and developed the sonography training, contributed to writing the protocol; DDW: Wrote the data analyses plan and reviewed the protocol. All authors read and approved the final manuscript.

## Pre-publication history

The pre-publication history for this paper can be accessed here:

http://www.biomedcentral.com/1471-2393/14/73/prepub
